# Do Doctors Differentiate Between Suicide and Physician-Assisted Death? A Qualitative Study into the Views of Psychiatrists and General Practitioners

**DOI:** 10.1007/s11013-020-09686-2

**Published:** 2020-08-24

**Authors:** Rosalie Pronk, Dick L. Willems, Suzanne van de Vathorst

**Affiliations:** 1 Department of General Practice, Medical Ethics Section, Academic Medical Centre, Amsterdam UMC, Amsterdam, The Netherlands; 2 Department of Medical Ethics and Philosophy, Erasmus Medical Centre, Rotterdam, The Netherlands; 3 Department of General Practice, Medical Ethics Section, Academic Medical Centre, Amsterdam UMC, Room J2-219, PO Box 22660, Amsterdam, The Netherlands

**Keywords:** Suicide, Physician-assisted death, Psychiatry, Psychiatric patients, Euthanasia, Netherlands

## Abstract

Physician-assisted death for patients suffering from psychiatric disorders is allowed in the Netherlands under certain circumstances. One of the central problems that arise with regard to this practice is the question of whether it is possible to distinguish between suicidality and a request for physician-assisted death. We set up this study to gain insight into how psychiatrists and general practitioners distinguish between suicidality and physician-assisted death. The data for this study were collected through qualitative interviews with 20 general practitioners and 17 psychiatrists in the Netherlands. From the interviews, we conclude that physicians distinguish three types of death wishes among patients suffering from psychiatric disorders: ‘impulsive suicidality,’ ‘chronic suicidality,’ and ‘rational death wishes.’ To discern between them they evaluate whether the death wish is seen as part of the psychopathology, whether it is consistent over time, and whether they consider it treatable. Some considered physician-assisted death an alternative to a ‘rational suicide,’ as this was perceived to be a more humane manner of death for the patient and their relatives. We argue that physician-assisted death can be justified also in some cases in which the death wish is part of the psychopathology, as the patient’s suffering can be unbearable and irremediable. Physician-assisted death in these cases may remain the only option left to relieve the suffering.

## Introduction

In the Netherlands, patients who suffer from psychiatric illnesses are not excluded from the practice of euthanasia and physician-assisted suicide (EAS) (the term ‘Medical Aid in Dying’ (MAID) is also used, in both Canada and the US. However, we will make use of the term ‘EAS,’ as this is how it is used in the Netherlands). The Dutch Supreme Court ruled in the Chabot case (in 1994) that the unbearableness of the suffering should be leading, regardless of the source of this suffering (Pans 2006). Physician-assisted death for patients suffering from psychiatric illnesses solely is also permitted under strict criteria in Belgium, Luxembourg, and Canada (Emanuel et al. 2016; Downie and Dembo 2016). In the Netherlands, a physician is allowed to provide assistance in dying if he or she meets the legal criteria of due care. These criteria hold that there should be a voluntary and well-considered request, the patient’s suffering should be unbearable and without the prospect of improvement, the patient should be informed about his or her situation, there are no reasonable alternatives to relieve suffering, an independent physician should be consulted, and the method used should be medically and technically appropriate (Regionale Toetsingscommissie Euthanasie 2018). The third evaluation of the Dutch euthanasia act, from 2016, shows that 88% of the general public supports the euthanasia law (Onwuteaka-Philipsen et al. 2017). However, EAS in case of psychiatric suffering is less accepted. Evenblij et al. showed that of the general public 53% though that people with psychiatric disorders should be eligible for EAS, 15% was opposed to this, and 32% remained neutral (Evenblij et al. 2019).

Dutch psychiatrists are generally very reluctant to perform EAS for psychiatric suffering. An important reason for their reluctance lies in the difficulty they have in determining the difference between a request for EAS and suicidality (Onwuteaka-Philipsen et al. 2017). Whether it is possible to distinguish irrational suicidality from rational death wishes matters because there is a societal commitment to suicide prevention (Miller and Appelbaum 2018). The general thought is that irrationally suicidal patients should not be eligible for EAS, as they require treatment for their psychopathology.

Psychiatrists view suicide as an act that is often the result of mental illness: those who want to commit suicide are not capable of making that decision due to their mental illness (Wittwer 2013). Some, however, argued that patients who suffer from a mental disorder can have a rational wish to die, for example, even in the case of schizophrenia (Hewitt 2010, 2013). The Dutch Association of Psychiatry (NVvP) takes a similar stance in their guideline on physician-assisted dying in the case of psychiatric suffering. On the one hand, they state that suicidality is often the result of underlying psychopathology. In those cases, the patient suffers from a psychiatric disorder that obstructs a clear judgment and therefore conflicts with decisional competence (Levensbeëindiging op verzoek bij patiënten met een psychische stoornis [End-of-life requests from patients with mental disorders] [Internet] 2018). On the other hand, the NVvP does not entirely reject the option of EAS, which implies that the NVvP also believes there is such a thing as a rational death wish. EAS could, however, only be acceptable if it could be established that the patient is suffering from a psychiatric illness, suffers unbearably without the prospect of improvement, and made a considered (rational) request.

In the Netherlands, both psychiatrists and general practitioners (GPs) respond to requests for EAS from patients who suffer from psychiatric illnesses (Euthanasie 2017). It is highly relevant how they view the relation between suicide and EAS, as they are the ones that assess the requests for EAS from these patients. We therefore conducted a qualitative study with 17 Dutch psychiatrists (as a part of the Third Evaluation of the Dutch Euthanasia Act) and 20 Dutch GPs on their views and considerations concerning EAS in psychiatry. We addressed the issue of EAS for persons suffering solely from psychiatric illnesses, and *not* from a combination of somatic and psychiatric illnesses. The relation between suicidality and EAS was one of the topics covered in the interviews. We chose to address this question by using a qualitative method, as this allowed physicians to elaborate on their views and considerations.

## Methods

### Design

The interviews with 17 Dutch psychiatrists were held as a part of the Third Evaluation of the Dutch Termination of Life on Request and Assisted Suicide Act. These interviews were held from January until June 2016. The interviews with the 20 Dutch GPs were held from September 2018 until February 2019. All interviews were held with the use of a topic list., Two pilot interviews were held to check whether the topic list was complete. The results presented in this paper are a part of a broader study on EAS among psychiatrists and general practitioners.

Two researchers conducted the interviews, RP and NS. RP performed all interviews with the psychiatrists, and 8 out of 20 interviews with GPs. NS conducted 12 of the interviews with Dutch GPs. To prevent bias, NS was trained by RP to do the interviews with the GPs, and she observed one of RP’s interviews. RP also observed one of NS’s interviews. They discussed these afterward to come to a mutual understanding about how to conduct the interviews. The interviews were held at the physician’s location of choice and lasted between one and 2 h. All physicians signed an informed consent form, which included statements on confidentiality and the voluntary character of participation.

### Respondents

The respondents were selected through purposive sampling. We aimed for a variety in experience with psychiatric EAS requests, views on psychiatric EAS, sex, subspecialty within psychiatry, and work environment. We included respondents who were in favor and against psychiatric EAS, who did and did not have experience with assessing and performing psychiatric EAS requests.

We recruited the psychiatrists at Expertisecentrum Euthanasie (previously: End-of-Life Clinic), through the professional network of the researchers, through snowball sampling and through the website of the Dutch patient federation. We recruited the GP’s through a questionnaire that was held under Dutch GPs, by addressing physicians following mental healthcare training, through the network of the Amsterdam UMC and snowball sampling.

### Data Analysis

We lost one interview with a psychiatrist and one interview with a GP due to technical problems, so a total of 16 interviews with psychiatrists and 19 interviews with GPs were included. All interviews were transcribed verbatim and inductively coded by RP. NS conducted 12 interviews with GPs. Three interviews with GPs were separately coded, compared, and discussed with NS. Five interviews with psychiatrists were also independently coded by a second coder (JG). We coded and created code trees to identify overarching themes. Data analysis and the results were discussed with the supervisors of the study (DW and SV). The results of the interviews with psychiatrists were also discussed within the research group that conducted the Third Evaluation of the Dutch Termination of Life on Request and Assisted Suicide Act.

## Results

### Three Categories of Death Wishes

Concerning patients who suffer from psychiatric disorders, most physicians distinguished between different types of death wishes, namely (a) impulsive suicidality, (b) chronic suicidality, and (c) a well-considered and persistent wish to die.

What you always do, in case of a psychiatric patient is evaluate how suicidal they are. And if you suspect they really are suicidal, you have to do something. Usually, you consult the experts, have them evaluated. But the fact that someone is suicidal, is something completely different than a euthanasia request (GP - R8)

That was one of the clients who was treated here, who had a persistent death wish that differed from the chronic suicidality that we often see with personality disorders. And the client indicated that she wanted to actively do something about that. (…) (psychiatrist - R13)

Impulsive suicidality (a) was characterized as a temporary wish to die, often related to emotional events, and a symptom of the psychopathology of the patient. Chronic suicidality (b) was characterized as being more persistent and frequently occurring, but still a symptom of the psychopathology of the patient. A rational death wish (c) was characterized as a well-considered persistent wish to die, unrelated to emotional events, and not an expression of the psychopathology. These patients carefully consider their situation and prospects and make an informed decision about whether they want to live or not.

However, there was also one respondent who did not believe there exists such a thing as a ‘rational death wish,’ as they indicated that all death wishes are the result of emotional distress:

(…) this is also one of my opinions: that rational suicide does not exist. That is the humanistic liberal thought that humans are rational beings, but I have never seen a rational suicide. All of this so-called weighing…(…) I have got a lot of experience, I have worked for 40 years in large hospitals, with large ERs and saw 3 cases of (i.e. attempted) suicide a week, for 40 years, so I have talked to a lot of them. A lot of the people who considered it carefully (i.e., suicide attempt), weighing the pros and cons…if you take the time to go through them, these are all hollow phrases. It is all emotions, it is all pain, anger, indignation, despair (psychiatrist - R8).

Multiple respondents mentioned that a death wish can be seen as a continuum, and suicidality is at a different position within that continuum than EAS is. Consistency over time was seen as a relevant factor in relation to that continuum:

I: I want to pick up on the death wish that is the result of a disease, let’s call that ‘suicidality’, and a long-lasting wish to die because someone thinks he/she suffers unbearably. What, to you, constitutes the difference, how do you separate those two?

R: Notably because of the phase that someone is at. So that depends on the clinical situation. (…) ‘time’ is an important factor, and where someone is. If someone, if you assess someone and there is no depression (…) and there still is a death wish, if that repeats itself during time, then you could say that it is not the result of a disease.. (GP - R13)

A man of 25 years old. What did he do? He went to the *name of the bridge*, and knotted a rope around it, around his neck, jumped (…) and the rope snapped and he went into the water, he wanted to commit suicide, he really tried (…). He swam to the side, got into the car and drove here [i.e. to the physician] (…). I think it is nice that he comes here for help, and then you start to do all kinds of things. But it shows how it goes with suicides. (…)‘This example shows impulsivity. A genuine euthanasia request is discussed during multiple conversations, that is consistent in general, that is completely different. (GP - R8)

However, some respondents did not believe such a continuum exists:

I always call that the ‘blackmail argument’. First of all, it is incorrect. Violent suicides are not an extension within a continuum of a well-considered gentle death, of a gentle suicide…towards a gruesome suicide. (…) There is no such thing as a continuum (…) But the thought that there is a continuum, there is no such thing. If you check the literature, it is a binomial distribution. So a small group of violent suicides, and a larger group of the repeated lighter [i.e. attempted] suicides. And they don’t mix. (psychiatrist - R8)

### Treatment for Suicidality

Respondents expressed that they believe it is their responsibility to treat patients and prevent deaths that are the result of impulsive suicidality and chronic suicidality (a, b), but not necessarily those that are the result of a rational death wish (c)

That does not mean that we have to accept all those impulse suicides, no, that would not be good. If somebody is suicidal, it is often a temporary state of mind. And after a few months, that person might look back onto that very differently, so you have to pull them away from that edge. (GP - R14)

[when the patient says]” I want this to happen when I’m really in touch with a medical professional, that would be the best. That is my deep inner wish. I am completely convinced about this, help me please, because if this does not happen, if I do not get what I want most, I will stagnate” Well, then it is difficult for me not to. (…) if this really is something authentic, why wouldn’t I help you? What stops me is that I would have to kill you, something that I really, deeply, do not want to. But if you would want to commit suicide…If you go with a blissful smile, dangling on the staircase of your choice, it is all as you wished, who am I to sort of comment on that? If it is in line with who you are. But the moment that I have the feeling that your reasoning is flawed, you tell me this in pain, this is not in line with other sides that I know of you, then I think it is my job as a therapist to confront you with that and not to say ‘you know what, okay’. (psychiatrist - R4)

GPs indicated that they find it complicated to assess the due care criteria for patients suffering from psychiatric illnesses: whether a patient has decisional competence, whether the death wish is well considered and without the prospect of improvement. Many GPs indicated that they prefer to work together with a psychiatrist:

I think that I would refer them to a psychiatrist with a clear question: are there any reasonable treatment options? That is a question that I would like to see answered. And also whether somebody is mentally competent. (GP - R15)

## Suicide or Physician-Assisted Death?

For some respondents, EAS could provide an alternative for a rational suicide. This respondent told us that he discusses the option of EAS with a patient if he has a long-lasting wish to die:

I mean, someone who thinks he can fly and jumps out of a window, I find that horrible, I want to prevent that. Someone who impulsively, because his relationship broke up, drinks too much, drives too fast and hits his car against a tree, I really would like to prevent that. But 10 years of treatment because of a psychotic disorder, having the prospect of never leading a normal life, and then having to jump off a high-rise building. I would sincerely hope he would request euthanasia, so I could offer a dignified and less lonely ending. And that rational suicide, I would like to translate that to euthanasia. (psychiatrist - R11)

Whereas some respondents thought EAS could serve as an alternative for suicide, various respondents expressed that they did not believe that EAS is a good alternative, for which they provided multiple reasons. The first reason related to the belief that the category of patients who commit suicide differs from the category of patients who request EAS:

No, I think that those who commit suicide, they might be more vigorous or don’t want any help with that, but they do not experience the threshold that people who request euthanasia do. It is a different, it is often a different population. (GP - R4)

Those numbers don’t go down because of self-euthanasia or euthanasia, because if that would have been the case, we should have seen that happening, with the doubling of the euthanasia cases in the past five years, the gruesome suicides have not reduced. That is because it is an entirely different population, you should not confuse those two.. (psychiatrist - R10)

Another reason provided was that it is not necessarily the case that people who do not have their EAS request granted, commit suicide:

No, I don’t think that at all. No, this is a misunderstanding. I think that it is definitely not the case that people who received euthanasia would otherwise have committed suicide, that is not the case. It might be the case sometimes, but not all times. The reasons for euthanasia and the reason for suicide might differ. It can be the case that you are very mad because of the rejection of your euthanasia request, and that you are taking revenge, (…) So, dying may have an interactional meaning, a communicative meaning instead of it being an answer to the big problem of ‘I cannot live with the pain I have’. So, those are two different lines of argument, that does not have to be about the same thing.. (psychiatrist - R4)

A third and final reason was that threatening to commit suicide to receive EAS can be a form of manipulation. This respondent indicated that he did not want to be at the receiving end of this manipulation and that he will turn to emergency care instead of providing EAS:

I would not let myself be forced. It cannot be the case that the patient is blackmailing me and says ‘if you don’t do it, I will do it myself’. I would never agree in such a situation. If I really believe that there is a big suicide risk, if the odds are really big, then that is a reason for me to get the emergency care involved. I would not think ‘oh, I will just agree with him because he is really serious’. I would not accept that. (GP - R15)

## What Do Physicians Perceive to be the Difference Between EAS and Suicide?

The respondents suggested multiple differences between suicide and EAS. We describe these differences under the following headings: (a) the causes and meaning of death wishes differ, (b) physician-assisted death as less traumatic, (c) treatability of the condition, and (d) consistency of the death wish and suffering.

### The Causes and Meaning of the Death Wish Differ

Respondents mentioned that they viewed suicidality as part of the psychiatric disorder, either an expression of it or directly caused by it, whereas an EAS request was considered an authentic expression of the person.

That seems wrong, because when somebody is suicidal. I was just saying (…) then that is the result of their psychiatric disorder, or a bad mood, or voices that someone hears, or irrational thoughts. While if someone has a persistent death wish, and I don’t think about a 20- or 30 year old, but when they have lived a long life, when it has been a long and hard road that they don’t want to take anymore, then it is not the result of a disorder, but it comes from a whole person who does not want to live anymore. (…). (GP - R17)

He also had a psychiatrist with whom he spoke, and I thought it was quite hard, so I asked that psychiatrist whether it was the result of depression or another psychiatric disorder. And he really analyzed it, and was able to say that this was a man who was always able to do things his way, it was really authentic for him, it fits, and that it was not a treatable psychiatric disorder. (GP - R7)

A second discerning factor was that suicidality was seen as an emotional reaction to a certain situation, in contrast to a well-considered wish to die:

R: That was with one of the clients that we have in treatment here, and at a certain moment there was a persistent death wish that was different from the chronic suicidality that we often see with personality disorders. This client indicated that she wanted to actively do something with that [i.e. her wish to die]. Then we decided to have a two-track approach, on the one hand, the application for Expertisecentrum Euthanasie, and on the other hand the treatment trajectory.

I: And if you say that the death wish was something different than chronic suicidality, what is the difference between those two. What constituted the difference?

R: I think that a difference is, chronic suicidality is something we see a lot, especially with borderline patients, but also with other personality disorders. It is often connected to emotion regulation problems, or emotional wounds. So, suicidal thoughts occur frequently, arise often, and can often be traced back to emotional wounds, in which someone justifiably or unjustifiably feels hurt by a circumstance, because of a reaction of someone. But there are also moments in which that does not occur. So you see that it occurs over the years, increases in some periods, so that they arise every week or even multiple times a week. But that there are also periods when they don’t occur, or occur less. With this client [i.e. who had a euthanasia request], she could not think about anything else, so it was really persistent. There were no good moments anymore, or a free moment, a moment where she would experience a will to live. (psychiatrist - R13)

Another respondent stated:

Yes, there is a big difference. People who for example are overwhelmed by heartbreak throw themselves in front of a train, hang themselves, take pills or slit their wrists. That is suicidality. I believe that you have to get them admitted, hold them, talk to them. And three weeks later, they’re better. And then they live happily ever after. That is something different than a deeply felt death wish in case someone has a persistent, chronic depression or anxiety disorder. (GP - R16)

As a third factor, respondents mentioned that suicidality can have a communicative meaning rather than actually wanting to end one’s life:

Look, most of these cases are a cry for help. Most suicide attempts are a cry for help. The real rational suicides, in which people have been weighing ‘do I really want this, or don’t I want this’ are far less common.. (psychiatrist - R13)

So if you have been neglected, never got something in life, and you want something, but the system is unwieldy and does not move, you can appeal to the system by speaking their language, which is risk-avoidant. The mental health care system is risk avoidant, so if you are saying ‘I’m going to commit suicide’, the system comes into action.. (psychiatrist R2)

Although respondents indicated that in their opinion the cause and meaning of impulsive suicidality differ from the cause and meaning in case of a request for EAS, they remarked that also not all EAS requests involve an authentic wish to die. Respondents described how a request for EAS might also have different meanings:

People are exploring how they can get their way, and yes, people with a lot of experience in the mental healthcare as a patient know that you can scare a psychiatrist with questions about life and death. They can assure themselves of special attention to that. But whether you should give that attention, or go along with that line of thinking, takes professional skill to find out. (psychiatrist R4)

That happened multiple times, that is the same process that I had in the elderly home, that it is more of a question for improvement of the quality of life and care and attention, than it is directed at the end of life. (psychiatrist R1)

It was indicated that most patients who are suicidal are ambivalent until the end, whereas someone with a consistent and well-considered death wish is not:

We know that about suicidal people. In the majority of cases, there is ambivalence until the end. And whether that is ambivalence (…). It can be that you have your reasons to want to die, but also have fear and are afraid of dying. With your reason you want to die, with your heart you are afraid. Those are two different instruments, you could say, that go against each other. So that is also ambivalence. (…) But you often see that people who are suicidal do things in very mixed states.. (psychiatrist R4)

### Physician-Assisted Death as Less Traumatic

Suicide was considered to be a complicated, violent, and lonely way to die:

If someone jumps in front of a train, that is an extremely harmful way to die, or don’t die, because I also saw someone whose legs got ripped off after jumping in front of a train. That was horrible. There is so much violence in it, it’s the violence that makes it so unpleasant. (GP - R17)

That you offer an alternative. Suicide is a very unpleasant intervention for the patient, for themselves, it is a very lonely road to take. (psychiatrist - R5)

EAS is, however, considered less traumatizing for the patient and his relatives, and it provides a possibility to say goodbye:

It (i.e. suicide) is horrible for the bereaved, they are left with a lot of questions and guilt. And it is also hard on the therapists. In short, it burdens the environment. And I think that euthanasia offers a better alternative for that.

I: Why?

R: Because you discuss the problem in the preliminary phase, the patient feels taken seriously, treatments are discussed seriously. Family is involved, in general. So, the end doesn’t come as a surprise. And there are no, in the best case, questions left and the goodbye can be experienced in a much better way. The grieving process, well, it precedes the euthanasia. So I think it is a more humane way to die than suicide.. (psychiatrist - R5)

### Treatability of the Condition

Another factor that distinguishes suicidality from EAS is the treatability of the condition. This respondent indicated that suicidality is considered treatable, while in the case of EAS the situation is without the prospect of improvement:

I: We were discussing the difference between suicidality and a lasting wish to die, how to differentiate and whether that difference is relevant.

R: Yes, it is very relevant, because it revolves around the question ‘is it without prospect of improvement’. Because with suicidality, as a part of the disease, it is by definition not untreatable, because it is treatable. Actually, that is very important, that if someone continues to be suicidal despite you having done everything you can, that distinction [i.e. between suicidality and a lasting wish to die] becomes relevant, because you have done everything you possibly can. (GP - R13)

### Consistency of the Death Wish and the Suffering

A final important difference was the fact that with EAS, the patient has a consistent death wish as a result of persistent suffering:

I: I believe I already asked, but what about the difference between suicidality and a lasting death wish, you would call in a psychiatrist?

R: Yes, I can imagine that I would do that. And, how consistent is someone, that is something I would find very important. And how much the image varies. If you talk to someone multiple times, maybe there are moments in which I think ‘Well, someone’s mood is pretty okay’, or it seems to go better. Or does someone stay very consistent in his suffering? (GP - R15)

## Discussion

We have conducted this study to gain insight into how Dutch psychiatrists and general practitioners differentiate between suicidality and EAS.

It seems that physicians distinguish between impulsive death wishes and considered death wishes. Impulsive death wishes are not seen as justified reasons for EAS and should be treated. Moreover, it matters whether the death wish is consistent over time and whether the physicians qualify the death wish as a symptom of the disease and therefore as a part of the psychopathology, or as a consequence of the disease (but not part of it), as is the case for somatic diseases. If the death wish is seen as part of the psychopathology, EAS will be less conceivable for the physicians. Another important factor is whether they think the death wish is treatable or not. If so, the patient is not seen as a candidate for EAS. Those whose death wish is seen as a consequence of their disease (but not a symptom of it), for instance, because they feel exhausted after having coped with their difficulties for years and years, and whose wish is irremediable, are seen as potential candidates for EAS. Their death wishes are compared to ‘balance suicides’, or ‘rational suicides’. The physicians indicated that the option of EAS offers a dignified ending: death by EAS is less traumatic for the patient and their environment.

These criteria tie in with the due care criteria in the Dutch euthanasia law, which demand that the death wish is well considered, meaning the patient does not merely have the capacity to choose, but is able to reflect on his wish to die. Two of the other criteria also seem to play a great role, and to be interconnected: the irremediableness and the unbearableness of the suffering.

Regarding the well-considered death wish, when patients use their death wish as a way to provoke attention from the therapist, or even as a form of blackmail, as an appeal to improve the quality of mental healthcare, or when it arises out of an emotional response, it is hard to see these as well-considered death wishes. These are, as commonly is said, ‘cries for help.’ Despite this, it is argued that wanting to end one’s life can be a rational choice also for psychiatric patients (Hewitt 2010, 2013; Berghmans 1992; Berghmans, Widdershoven, Widdershoven-Heerding 2013). The decisional capacity is not affected all the time in all patients; some may have periods in which their capacity for understanding, reasoning, and communicating is intact. We believe that although certain psychiatric disorders can increase the risk of incapacity, this does not mean that all patients with psychiatric disorders should be considered incapable of making rational choices concerning their own death (Doernberg, Peteet, Kim 2016).

The other two criteria for EAS in the Dutch law relate to the suffering that the patient experiences, and demand that it is without the prospect of improvement and unbearable. This of course excludes patients for whom treatment is expected to improve their suffering. This is completely in line with what the interviewees stated, that if there is still the possibility of improvement, EAS should not be an option.

However, there also seems to be a group of patients whose death wish may well be part of their disease and at the same time the cause of their irremediable suffering (i.e., the chronically suicidal patients). In these cases, physicians seem to struggle with judging how voluntary and well considered their death wish is, i.e., what the influence of the psychopathology is. Even if the death wish is part of the psychopathology, the patient is heavily burdened. These patients suffer extensively as a result of their psychopathology and from their persisting wish to die. It is the chronicity of the wish to die that is an important part of the unbearable suffering, and it is unsurprising that physicians struggle with this group.

We wondered whether EAS can be a justifiable option in the case a patient is chronically suicidal, suffers unbearably, and without the prospect of improvement, but cannot reflect well enough upon his death wish anymore because it is so entangled with his psychopathology? It is clear the physicians struggle with this group. First of all, we need to make clear that the patient does need to request EAS, we are not discussing a category of patients who cannot make a request due to their condition or do not want EAS. The question then is whether it is acceptable to lower the threshold for the ‘well-considered and voluntary’ criterion in some cases. The suffering of these patients is undeniable, and after several (or even many) failed treatments can be considered as untreatable. If we stick to the demand that the patient should be able to reflect on his death wish, and that the death wish should be well considered, EAS would not be possible. However, if the patient clearly suffers so unbearably *and* their situation is without treatment options, we suggest it is worth considering that the physician may plead *force majeure* as he or she can have good reasons to perform EAS, because it is the only option left to end the patient’s suffering. Although controversial, we would think this could provide a humane solution for patients who suffer unbearably and who are without the prospect of improvement from psychiatric illnesses.

## Strengths and Limitations

To our knowledge, this is the first qualitative study that covers the question about how physicians view the relation between suicide and physician-assisted death. In-depth insight into this topic is of meaning for the debate around EAS, as it is an important consideration for doctors with regard to the acceptability of EAS. A limitation of this paper is the fact that the relation between suicidality and physician-assisted death was only one of the topics covered in the interviews, and not the main focus of an entire study. This could have led to a limitation in answers.

Another limitation is the fact that the interviews were held by two interviewers, RP and NS. Although NS was trained by RP to do the interviews, this could have led to a difference in questioning.

## References

[CR1] Berghmans R. 1992 Om bestwil. Paternalisme in de psychiatrie [In your best interest. Paternalism in psychiatry]. Amsterdam: Thesis Publishers.

[CR2] Berghmans R., Widdershoven G., Widdershoven-Heerding I. (2013). Physician-Ussisted Suicide in Psychiatry and Loss of Hope. International Journal of Law and Psychiatry..

[CR3] Doernberg S.N., Peteet J.R., Kim S.Y. (2016). Capacity Evaluations of Psychiatric Patients Requesting Assisted Death in the Netherlands. Psychosomatics..

[CR4] Downie J., and J. Dembo 2016. Medical Assistance in Dying and Mental Illness Under the new Canadian Law. Journal of Ethics in Mental Health. 1.

[CR5] Emanuel E.J., Onwuteaka-Philipsen B.D., Urwin J.W., Cohen J. (2016). Attitudes and Practices of Euthanasia and Physician-Assisted Suicide in the United States, Canada, and Europe. JAMA..

[CR6] Euthanasie, R. T. and Jaarverslag 2017 [Annual Report] Den Haag2018.

[CR7] Evenblij K., Pasman H.R.W., van der Heide A., van Delden J.J., Onwuteaka-Philipsen B.D. (2019). Public and Physicians’ Support for Euthanasia in People Suffering from Psychiatric Disorders: A Cross-sectional Survey Study. BMC Medical Ethics..

[CR8] Hewitt J. (2010). Schizophrenia, Mental Capacity, and Rational Suicide. Theoretical Medicine and Bioethics..

[CR9] Hewitt J. (2013). Why are People with Mental Illness Excluded from the Rational Suicide Debate?. International Journal of Law and Psychiatry..

[CR10] Levensbeëindiging op verzoek bij patiënten met een psychische stoornis [End of life requests from patients with mental disorders] [Internet]. 2018 Available from: https://richtlijnendatabase.nl/richtlijn/levensbeeindiging_op_verzoek_psychiatrie/startpagina_-_levensbe_indiging_op_verzoek.html.

[CR11] Miller F.G., Appelbaum P.S. (2018). Physician-Assisted Death for Psychiatric Patients - Misguided Public Policy. The New England Journal of Medicine..

[CR12] Onwuteaka-Philipsen, B., J. Legemaate, A. van der Heide, H. van Delden, K. Evenblij, I. El Hammoud, R. Pasman, C. Ploem, R. Pronk, S. van de Vathorst, and D. Willems 2017 Derde evaluatie Wet toetsing Levensbeeindiging op verzoek en hulp bij zelfdoding [Third Evaluation of the Termination of Life on Request and Assisted Suicide Act] Reeks evaluatie regelgeving. 16223080

[CR13] Pans E. 2006 De normatieve grondslagen van het Nederlandse euthanasierecht. [Normative foundations of the Dutch Euthanasialaw] Tilburg: Wolf legal publishers: Vrije Universiteit Amsterdam

[CR14] Regionale Toetsingscommissie Euthanasie. EuthanasieCode 2018 Den Haag2018.

[CR15] Wittwer H. (2013). The Problem of the Possible Rationality of Suicide and the Ethics of Physician-Assisted Suicide. International Journal of Law and Psychiatry..

